# Monitoring ferumoxide-labelled neural progenitor cells and lesion evolution by magnetic resonance imaging in a model of cell transplantation in cerebral ischaemia

**DOI:** 10.12688/f1000research.2-252.v2

**Published:** 2014-03-04

**Authors:** Rachael A Panizzo, David G Gadian, Jane C Sowden, Jack A Wells, Mark F Lythgoe, Patrizia Ferretti

**Affiliations:** 1 Developmental Biology Unit, UCL Institute of Child Health, University College London, London, WC1N 1EH, UK; 2 Imaging and Biophysics Unit, UCL Institute of Child Health, University College London, London, WC1N 1EH, UK; 3 UCL Centre for Advanced Biomedical Imaging, Department of Medicine, University College London, London, WC1E 6DD, UK

## Abstract

Efficacy of neural stem/progenitor cell (NPC) therapies after cerebral ischaemia could be better evaluated by monitoring *in vivo* migration and distribution of cells post-engraftment in parallel with analysis of lesion volume and functional recovery. Magnetic resonance imaging (MRI) is ideally placed to achieve this, but still poses several challenges. We show that combining the ferumoxide MRI contrast agent Endorem with protamine sulphate (FePro) improves iron oxide uptake in cells compared to Endorem alone and is non-toxic. Hence FePro complex is a better contrast agent than Endorem for monitoring NPCs. FePro complex-labelled NPCs proliferated and differentiated normally *in vitro*, and upon grafting into the brain 48 hours post-ischaemia they were detected *in vivo* by MRI. Imaging over four weeks showed the development of a confounding endogenous hypointense contrast evolution at later timepoints within the lesioned tissue. This was at least partly due to accumulation within the lesion of macrophages and endogenous iron. Neither significant NPC migration, assessed by MRI and histologically, nor a reduction in the ischaemic lesion volume was observed in NPC-grafted brains.  Crucially, while MRI provides reliable information on engrafted cell location early after an ischaemic insult, pathophysiological changes to ischaemic lesions can interfere with cellular imaging at later timepoints.

## Introduction

Stroke remains a common cause of mortality and disability worldwide, with few effective treatments available. Cerebral ischaemia, caused by the reduction of blood flow in a cerebral artery, is the most common type of stroke, accounting for 80% of strokes ^1^. Cellular therapy is an emerging and promising avenue for treatment of cerebral ischaemia. Several experimental studies have demonstrated that treatment with neural stem cells after an ischaemic event improves neurological score and behavioural recovery, and can reduce infarct volume ^2– 5^. These findings have led to a small number of clinical trials of cellular therapy in stroke patients, with modest effects on neurological outcome and motor skill recovery ^6– 9^. It is clear that further optimisation of cell type and cell delivery, and a greater understanding of the behaviour of engrafted cells *in vivo* is required for improved therapeutic outcome.

Previous research has demonstrated that fetal telencephalic neural progenitor cells engrafted into the ischaemic brain are capable of migration towards the infarct, and of improving behavioural recovery ^10^. A small percentage of engrafted neural stem/progenitor cells (NPCs) have been shown to differentiate into neurons in the peri-infarct area and integrate into the neural network ^11– 18^. Developmentally, NPCs give rise to the cortical neuron layers in the normal developing embryo, and may have the appropriate migratory capacity and neurogenic potential to give rise to neurons after engraftment into host injury tissue.

*In vivo* monitoring of engrafted cells is an important tool that could be developed to correlate cell behaviour and distribution with clinical outcome. Cellular imaging with MRI contrast agents can be used to monitor the distribution of transplanted cells in models of cerebral ischaemia. Hoehn *et al.* demonstrated that mouse embryonic stem (ES) cells could be labelled with an iron oxide-based agent ^19^ and subsequently migrate into the infarcted area from the contralateral hemisphere. Cell migration and functional improvement has been observed in rat models of stroke after intracisternal injection of superparamagnetic iron oxide (SPIO)-labelled neurospheres ^20^. Engrafted cells labelled with SPIO have been shown to survive and differentiate into neural lineages in the ischaemic striatum ^21^.

Further development of contrast agents with low toxicity, high labelling efficiency and retention is required for improved detectability and effective long-term cell tracking *in vivo*.

The ferumoxide Endorem is a dextran-coated superparamagnetic iron oxide agent that can form a complex with the polycationic protamine sulphate – the FePro complex ^22– 24^. Previous studies have suggested that the FePro complex has little effect on cell viability and behaviour ^23– 25^.

Therefore we wished to assess the effect of FePro on NPC behaviour, and establish whether labelling and *in vivo* imaging of NPCs could be enhanced by using this complex. We studied the effect of FePro labelling on NPC metabolism, proliferation and differentiation capacity. FePro-labelled NPCs were engrafted into the ipsilateral parenchyma at 48 hours post-ischaemia and monitored over 4 weeks using MRI. Assessment of NPC proliferation and differentiation capacity demonstrated no difference in the behaviour of FePro-labelled NPC compared to unlabelled NPC. *In vivo*, we observed the evolution of hypointense T _2_ contrast in the ischaemic striatum over several weeks in both control and FePro-NPC engrafted animals.

## Materials and methods

### Neural stem cell cultures

For initial cell labelling assays and quantification of iron, the ST14A rat neural progenitor cell (NPC) line was used, and cultured as previously described (generous gift from E. Cattaneo) ^26^. For subsequent labelling, viability, proliferation and differentiation assays, and for the *in vivo* study, primary embryonic forebrain-derived NPCs were used.

Forebrain tissue was dissected from E13.5 CD1 mouse embryos (Charles Rivers UK) and digested in Trypsin with DNase. Cells were washed in trypsin inhibitor and Earl’s Balanced Salt Solution (EBSS), and plated in 6 well culture dishes. Proliferation medium contained DMEM:F12 with glutamax (Invitrogen), N2 supplement (GIBCO), 10 ng/ml EGF (Peprotech), 20 ng/ml FGF-2 (Peprotech), 0.05% heparin and 1% Penicillin-Streptomycin. Cells were incubated at 37°C with 5% CO _2_. Under these conditions neural stem/progenitor cultures (NPC) form aggregates called neurospheres that grow in suspension. Neurospheres were passaged every 7 days. The ST14A rat neural progenitor cell line was cultured as previously described ^26^. All reagents were from Sigma, unless otherwise stated.

### Cell labelling with contrast agent

For neurosphere labelling, neurospheres were dissociated at 6 days after passage, by incubation in 1X trypsin with DNase for 7 minutes at 37°C. Cells were washed in trypsin inhibitor and EBSS, and then incubated with contrast agent (Endorem or the FePro complex) in proliferation medium for 24 hours to allow contrast agent internalisation. Cells were washed in EBSS, and dissociated for replating or use in intracerebral microinjections.

For adherent ST14A cell labelling, cells were incubated with contrast agent in culture medium for 24 hours to allow contrast agent internalisation, washed three times with EBSS, then passaged.

*Endorem:* Cells were incubated with 500 µg/ml Endorem (Guerbet Laboratories Ltd, UK).

*FePro complex:* 2 mg/ml protamine sulphate was incubated with Endorem in a ratio of 9:5 for 10 minutes at room temperature to form the complex (FePro), making a final concentration of 100 µg/ml iron and 10 µg/ml protamine sulphate in the culture medium.

### Cellular viability assays

Cells were incubated with Endorem, FePro, or no contrast agent (control) for 24 hours. Cells were washed and replated into 96 well plates at a density of 10 ^4^ cells per well, 8 wells per treatment group, for cell metabolic activity assays as a measure of cell viability. The MTT (3-[4,5-dimethylthiazol-2-yl]-2,5-diphenyl tetrazolium bromide; Sigma) and Alamar Blue (Invitrogen) assays were performed as previously described ^27, 28^. For the Trypan Blue assay, cells were resuspended 10:1 in 0.5% Trypan Blue (Sigma), and transferred to a haemocytometer for counting. Under a light microscope (Zeiss), non-viable blue-stained cells and total viable cells were counted. For each assay, two samples were counted for each treatment group, and the assay was repeated three times.

### Cellular proliferation assays

The neurosphere-forming assays measured the number of neurospheres generated per 1000 cells and the average neurosphere diameter per sample, which are indicators of the number of stem and progenitor cells in the sample and their rate of proliferation, respectively. Cells were plated at a density of 10 ^4^ cells per ml in neurosphere proliferation medium into 96 well plates, where each well contained 100 µl of proliferation medium and 10 ^3^ cells. After 5 days in culture, the number of neurospheres generated per 10 ^3^ cells was counted, and neurosphere diameter was measured using the imaging analysis software, ImageJ developed by U. S. National Institutes of Health, Bethesda, Maryland, USA ( http://imagej.nih.gov/ij/, 1997–2012). For each assay, a minimum of 8 wells per treatment group was counted, and each assay was repeated twice.

### Prussian blue stain for iron in neurospheres

NPCs cultured at a density of 10 ^4^ cells per ml for 7 days were transferred to polyornithine-coated coverslips in 24 well plates for one hour to allow neurosphere adherence to coverslips. NPC were then fixed in 4% paraformaldehyde (PFA) in PBS for 10 minutes, incubated in 6% hydrochloric acid and 4% potassium ferrocyanide for 20 minutes, then washed in PBS and mounted.

### Cellular differentiation assays

Neurospheres at 6 days after passage were differentiated as previously described ^29^. Differentiated neurospheres were fixed in 4% PFA at 4°C. For immunocytochemistry, coverslips were incubated in blocking solution (10% goat serum with 0.1% Triton-X) for 30 minutes, then incubated in primary antibody in blocking solution for one hour at 37°C: IgG _1_ monoclonal mouse anti-β3-tubulin (1:1000, Promega G7121: G-purified monoclonal antibody from clone 5G8 raised in mice against a peptide (EAQGPK) corresponding to the C-terminus of β3-tubulin; the antibody has been tested to perform in frozen and paraffin-embedded sections of rat brain, cerebellum and spinal cord, human and rat fetal CNS progenitor cell cultures and adult human paraffin-embedded brain); polyclonal rabbit anti-GFAP (1:1000, Chemicon AB5804; raised in rabbit against purified bovine GFAP; routinely evaluated by immunohistochemistry on brain tissue, astrocytes, and neurons by Chemicon); monoclonal O4 IgM (1:2, generous gift Prof Rhona Mirsky) ^30, 31^. Coverslips were washed three times in PBS, then incubated with secondary antibody for 30 minutes at 37°C. Secondary antibodies were: Cy3-conjugated goat anti-mouse (1:100, Invitrogen A10521); Alexa 488-conjugated goat anti-rabbit 1gG (1:1000, Invitrogen A11008); and Alexa 680-conjugated goat anti-mouse IgM (1:1000, Invitrogen A21048). Coverslips were washed in PBS and mounted onto slides for fluorescence imaging.

### Measurement of iron using a superconducting quantum interference device (SQUID)

ST14A cells were labelled with Endorem or FePro using the labelling methods described above. Labelled cells were then washed and fixed in 4% PFA. Cells were transferred to gelatin capsules (size 0, Capsugel) containing cotton wool, at a density of 1.25 × 10 ^6^ per gel cap. Iron content of samples was measured using a superconducting quantum interference device (SQUID) and the amount of iron per cell was calculated. Sample magnetization was measured over a range of magnetic field strengths at 300K (Kelvin; room temperature) and 10K, and hysteresis loops were plotted. To calculate the amount of iron in each sample, remnant magnetization and saturation magnetization of the samples were compared to the magnetization of a sample with Endorem alone, which had a known iron content against which cell magnetisation was calibrated.

### Middle cerebral artery (MCA) occlusion and intracerebral microinjections

All procedures were carried out under the Animals Scientific Procedures Act 1986 (project license: PPL 70/05617). Eleven wild type male Sprague-Dawley rats (250 g, 8 weeks old, Charles Rivers, UK; n=11) were anaesthetised with 2% isofluorane. Temperature was monitored with a rectal probe. The middle cerebral artery occlusion was performed as previously described ^32^, to occlude the MCA for 30 minutes. Briefly, the right common carotid artery was exposed. A sterile 4-0 suture with an epoxy resin tip (Araldite ^®^, Huntsman Advanced Materials) was inserted into the carotid artery and advanced 17 mm into the brain to occlude the MCA for 30 minutes. Following occlusion, the MCA was reperfused by slowly removing the suture, and animals were then recovered on a heated mat. Animal weight was monitored. Following MCAO surgery, animals were assigned a number and alternately allocated to control or treatment group. After surgery, animals were housed individually, provided with soft tissue bedding and had unrestricted access to water and softened food pellets.

In six animals, FePro-labelled NPCs (FePro-NPCs) were injected into the ipsilateral corpus callosum 48 hours following cerebral ischaemia. Animals were anaesthetised with 2% isofluorane, and secured in a stereotactic frame (Kopf, Germany). Animals were injected with 1.25 × 10 ^5^ FePro-labelled cells in a maximum of 3 µl, as described above, at 0.2 l/min into the ipsilateral corpus callosum. The coordinates used were AP +1.0, ML -0.3; DV -0.2 to Bregma. Five animals received no intracerebral injection (control uninjected group).

Animals were imaged to determine whether injected cells could be identified and monitored over time with MRI, relative to the control uninjected group.

### MRI

For *in vitro* MRI, contrast agent-labelled cells were washed and fixed in 4% PFA. Cells were transferred to 250 µl Eppendorf tubes and centrifuged to produce a cell pellet. Eppendorfs were placed in a custom-made probe and imaged using a 2.35T horizontal bore magnet (Oxford Instruments, UK) interfaced to an SMIS console (Guildford, UK). A 2D spin echo sequence was used with the following parameters: TR=1500 ms; TE=80 ms; FOV 25 mm; slice thickness 1 mm.

For *in vivo* MRI, animals were anaesthetised with 2% isofluorane, and secured on a stereotactic probe. Animals were imaged using the above 2.35T system. A 2D gradient echo (T _2_*-weighted) sequence was used with the following parameters: TR=500 ms; TE=30 ms; FOV 30 mm; 128 × 128 voxels; 11 slices; 1 mm slice thickness; and 16 averages. A 2D spin echo (T _2_-weighted) sequence was also used: TR=1500 ms; TE=120 ms; FOV 30 mm; 128 × 128 voxels; 7 slices; slice thickness 1 mm; 16 averages. Animals were imaged at 3, 7, 14, 21, and 28 days post MCA occlusion surgery. For lesion volume measurements, hyperintense lesion areas from T _2_-weighted images were segmented manually using ImageJ software available at http://rsb.info.nih.gov/ij (developed by Wayne Rasband, National Institutes of Health, Bethesda, MD). Lesion areas per slice were used to determine the total brain lesion volume, taking into account the thickness of the 2D slices (total brain lesions covered a mean of 7.33 (1.44 SD) slices in FePro-NPC animals and 7.50 (1.27 SD) slices in control animals). The lesion volume of the central slice covering the central MCA territory was measured and compared between groups. Homologous regions of interest were defined in the contralateral hemisphere.

### Histology

After the final MRI time point, animals were euthanized by transcardial perfusion with 0.9% saline then perfusion fixed with 4% PFA. Brains were removed from the skull and transferred to 30% sucrose in PBS for 48 hours, then frozen and stored at -80°C for tissue processing.

Coronal brain sections of 30 μm thickness were taken on a cryostat (Bright Instruments, UK). For Prussian blue staining, sections were incubated in 6% hydrochloric acid and 4% potassium ferrocyanide for 20 minutes, then washed in PBS. Brain sections were counterstained with nuclear fast red or hematoxylin-eosin, and mounted with DPX (distyrene, plasticizer, xylene) mountant.

### Neurosphere migration assay

The MCA occlusion surgery was performed and animals were imaged at 24 hours post-ischaemia to visualize the infarcted area. Four animals underwent MCA occlusion surgery and were euthanised using Schedule 1 method at 24 hours (n=2) or 48 hours (n=2) post-ischaemia. The area of infarct, as determined by MRI at 24 hours post-ischaemia, was isolated. The contralateral hemisphere was used as non-ischaemic control tissue. Tissue was homogenized and filtered through a 0.4 μm sterile filter. Protein content of the homogenate was calculated against an albumin standard using the Pierce BCA Protein Assay Kit.

Single neurospheres were transferred into polyornithine-coated wells of 96 well plates, in 100 μl of culture medium per well. Individual neurospheres were photographed on a Zeiss AxioVert inverted microscope with a Hamamatsu digital camera and using OpenLab imaging software, then tissue homogenate or growth factors were added to the control medium. Treatment groups were as follows: 1400, 700, 300 μg/ml MCAO homogenate from 24 hours time point; 1400, 700, 300 μg/ml MCAO homogenate from 48 hours time point. Control groups were as follows: DMEM:F12 with N2 medium; DMEM:F12 with control tissue homogenate. Neurospheres were photographed 72 hours following treatment and the area covered by the cells measured to quantify the extent of migration. Assays were repeated three times, with 8 wells per treatment group.

### Data analysis

Data are expressed as mean +/- standard error. Data analysis was performed using ImageJ for MRI image analysis and neurosphere diameter measurements; Adobe Photoshop for microscopy image analysis, and SPSS software for statistical analysis. A one-way ANOVA was used for statistical comparisons, with P<0.05 defined as significant. A two-way mixed ANOVA was used for statistical analysis of the change in lesion volume over time in control and FePro-NPC-treated animals.

## Results

### Neurosphere response to cerebral ischaemia

**Figure 1.**
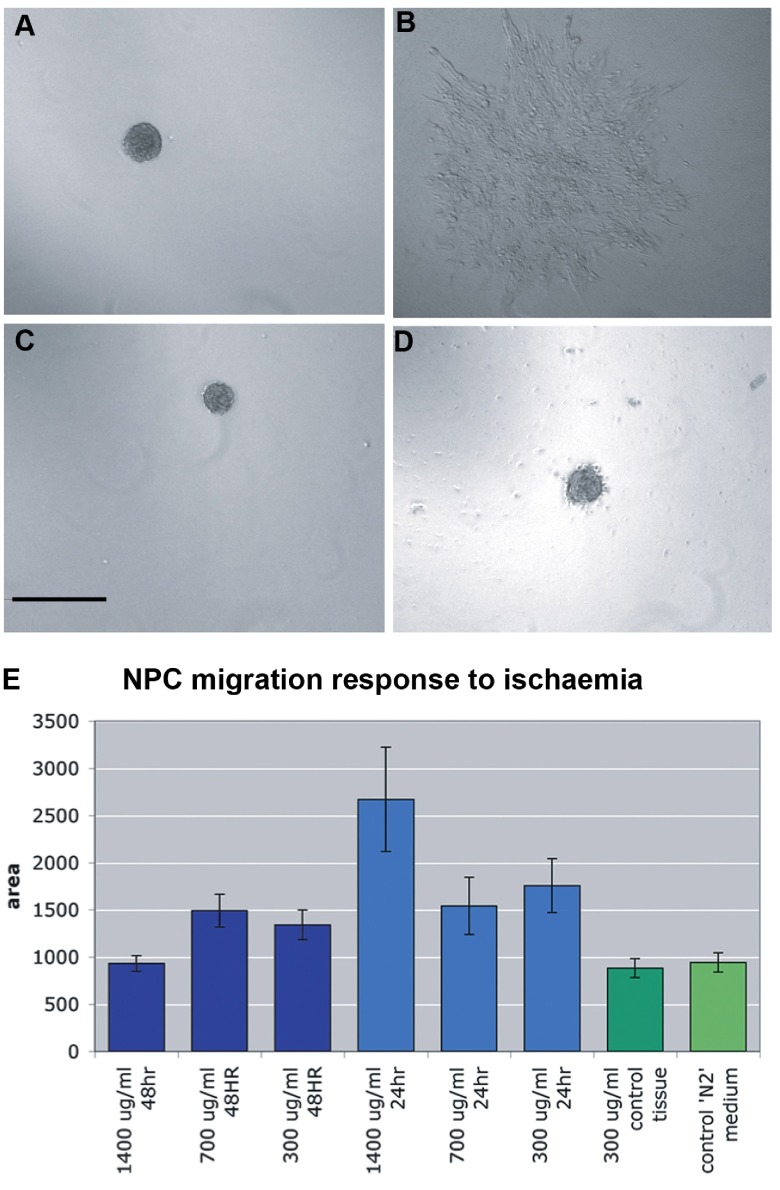
NPC neurosphere response to cerebral ischaemia. **A**, **B**, Migration of individual neurosphere in response to 1400 μg/ml protein from 24 hour MCAO homogenate at t=0 hr ( **A**) and t=72 hr ( **B**). **C**, **D**, Migration of individual neurosphere in response to contralateral hemisphere MCAO proteins at t=0 hr ( **C**) and t=72 hr ( **D**). **E**, Cell migration from E14 neurospheres at 72 hours with no proteins or treatment with proteins from either normal brain homogenates or from 24 (light blue bars) and 48 (dark blue bars) hours MCAO homogenates at different concentrations; *: p<0.001. Scale bar, 400 µm.

We assessed whether the NPCs expanded as neurospheres from embryonic mouse forebrain were able to respond to cues from cerebral ischaemia. We investigated their migratory response to protein extracted from cerebral ischaemia infarct regions at two different timepoints – 24 and 48 hours post-ischaemia and from the corresponding contralateral control ( Figure 1). The protein extracts were added directly to the wells containing the individual neurospheres; hence migratory cues were evenly distributed.

As shown in Figure 1A–E, extensive migration in all directions had occurred by 72 hours from neurospheres exposed to ischaemia protein extracts ( Figure 1A,B) but not in the presence of control proteins ( Figure 1C,D). Figure 1E demonstrates the migratory response of NPC to 24 hr and 48 hours ischaemic tissue protein extracts at different protein concentrations. Migration in both 24 hours and 48 hours post-ischaemia groups was significantly different from control migration (F=6.680; p<0.001). Post hoc analysis showed that at 24 hr, the 1400 µg/ml concentration, and at 48 hours the 300 and 700 μg/ml MCAO concentrations, were significantly different from control non-ischaemic tissue protein extract. Hence NPCs are able to respond to cerebral ischaemia cues *in vitro*.

Rat neurosphere response to cerebral ischaemia Neurosphere migration assay. Cell migration (um2) from E14 neurospheres at 72 hours with no proteins (N2 medium) or treatment with proteins from either normal rat brain homogenate (control tissue) or from different concentrations of 24 and 48 hours post middle cerebral artery occlusion homogenates.

### FePro cell labelling and viability

**Figure S1.**
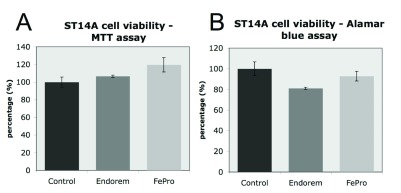
Effect of FePro- and Endorem-labelling on ST14A viability, and growth. **A**, MTT assay. No decrease in the percentage of viable ST14A cells is observed between control, Endorem-, and FePro-labelled cells after 24 hour treatment with MRI contrast agents; a small increase is observed in FePro-labelled cells (p<0.05). **B**, Alamar Blue assay. No significant difference is observed between FePro-labelled and control unlabelled cells, but there is a decrease in metabolic activity of Endorem-labelled cells relative to control unlabelled cells (p<0.01).

The effects of iron oxide-based MRI contrast agents on cell viability were first assessed in a fetal neural stem cell line and on primary NPCs. Incubation of ST14A cells with Endorem or FePro for 24 hours did not affect cell viability as assessed using metabolic activity assays ( Supplementary Figure 1). Iron content was measured using a SQUID. Mean iron uptake was 3.93 pg Fe/cell for Endorem-labelled cells, and 14.5 pg Fe/cell for FePro-labelled cells. Therefore the iron content was 3.7-fold greater in FePro-labelled cells than in Endorem-labelled cells. The efficiency of uptake of the FePro label into cells was not precisely quantified using Prussian Blue staining method, however high FePro accumulation was easily observed in at least 50% of the cells incubated with the contrast agent.

**Figure 2.**
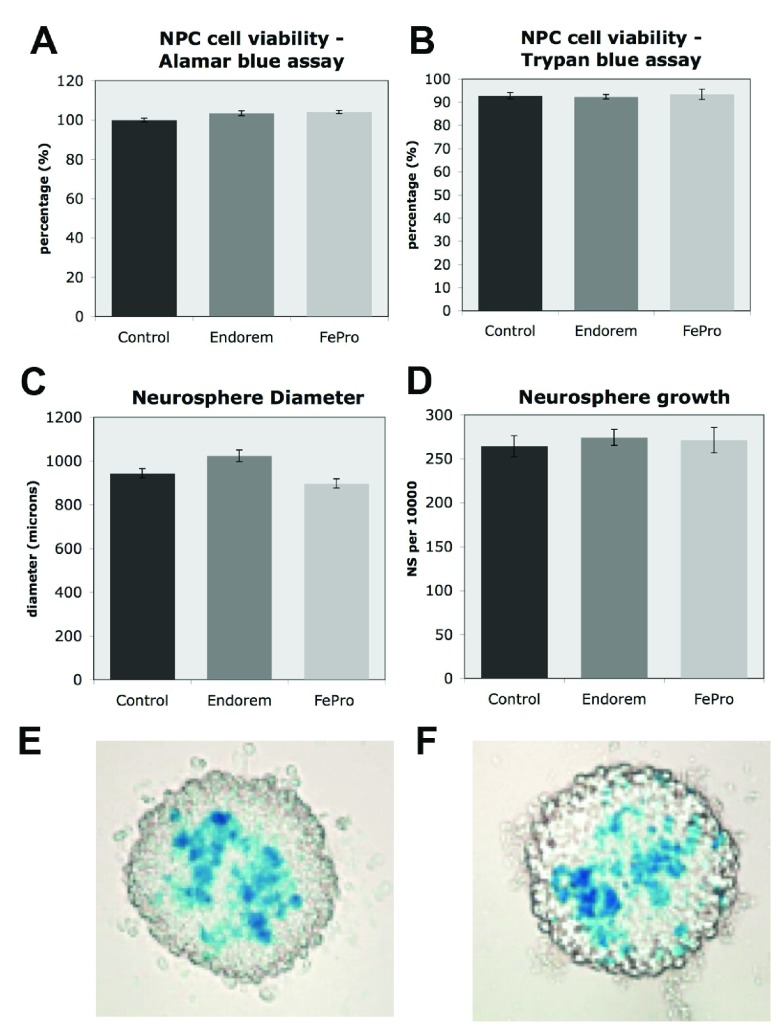
Effect of FePro- and Endorem-labelling on NPC viability and growth. **A**, Alamar Blue assay. No significant difference in NPC metabolism (F=0.736, p=0.49) is observed. **B**, Trypan Blue assay. No significant difference in cell survival (F=0.106, p=0.901) is observed. **C**, Neurosphere diameter. Diameter (µm) of Endorem-labelled neurospheres is significantly different from control unlabelled neurospheres (F=7.497; p<0.05). **D**, Neurosphere forming ability. No significant difference in neurosphere formation per 1000 cells (F=0.184; p=0.833) is observed. **E**, **F**. Prussian Blue stain for iron in Endorem (G) and FePro (H) labelled neurospheres after 7 days in culture.

We then investigated the effect of cell labelling with FePro and Endorem on NPC viability and proliferative capacity ( Figure 2A–D). Neither contrast agent affected NPC viability as shown by the Trypan Blue exclusion assay and Alamar blue assay ( Figure 2A,B). Furthermore, neither neurosphere-forming ability nor neurosphere growth were negatively affected by FePro or Endorem ( Figure 2C,D).

The iron within NPC neurospheres was visualized after 7 days in culture by Prussian blue. As shown in Figure 2E,F, the contrast agent is retained within cells across cell divisions.

**Figure 3.**
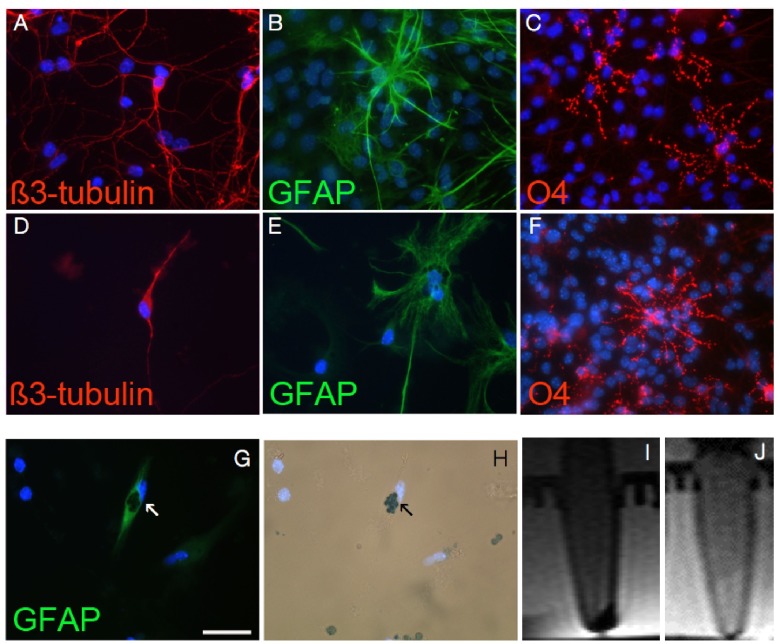
Differentiation capacity of FePro-labelled NPC. **A**– **F**. Immunocytochemistry of FePro ( **A**– **C**) and Endorem ( **D**– **F**) labelled neurospheres. **A**, **D**, anti-β3-tubulin (neuron, red). **B**, **E**, anti-GFAP (astrocyte, green). **C**, **F**, anti-O4 (oligodendrocyte, red). Blue, Hoescht nuclear stain. FePro-labelled neurospheres can differentiate into neurons ( **A**, **D**), astrocytes ( **B**, **E**), and oligodendrocytes ( **C**, **F**). **G**, immunocytochemistry of FePro-labelled astrocyte (GFAP, green). **H**, corresponding light micrograph showing Prussian blue stain. **I**, **J** MRI T _2_-weighted image of FePro-labelled neurosphere pellet ( **I**) and control unlabelled cell pellet ( **J**). FePro-labelled cell pellets produce hypointensity on MRI images. Arrows denote a GFAP-labelled cell co-labelled with Prussian blue, indicating that the FePro-labelled cell was capable of astrocyte differentiation. Scale bars, 20 µm.

To further characterise the effects of Endorem and FePro, the differentiation capacity of contrast agent-labelled NPCs was assessed. Figure 3 shows that FePro and Endorem-labelled NPCs can differentiate into neurons, oligodendrocytes, and astrocytes ( Figure 3A–F). The cells retain the contrast agent label after differentiation ( Figure 3G,H) and FePro-labelled cells generate negative contrast on MRI ( Figure 3I,J).

Effect of FePro- and Endorem-labelling on rat ST14A neural progenitor cell and neurosphere viability and growth in response to ischaemia in vitro MTT assay file legend: MTT ((3-[4,5-dimethylthiazol-2-yl]-2,5-diphenyl tetrazolium bromide) colorimetric viability assay of ST14A rat neural progenitor cells treated with Endorem or FePro MRI contrasting agents compared to control. Values are absorbance at 560nm.
Alamar blue ST14A legend: Alamar blue colorimetric viability assay of ST14A rat neural progenitor cells treated with Endorem or FePro MRI contrasting agents compared to control. Values are absorbance at 560nm.
Alamar blue NPC legend: Alamar blue colorimetric viability assay of rat neurospheres treated with Endorem or FePro MRI contrasting agents compared to control. Values are absorbance at 560nm.
Trypan blue NPC legend: Trypan blue viability assay of rat neurospheres treated with Endorem or FePro MRI contrasting agents compared to control. Number of viable cells is given as a percentage.
Neuosphere diameter legend: Neurosphere diameter assay to assess cellular proliferation of rat neurospheres treated with Endorem or FePro MRI contrasting agents compared to control. Values given in um2.
Neuosphere growth legend: Neurosphere growth assay to assess cellular proliferation of rat neurospheres treated with Endorem or FePro MRI contrasting agents compared to control. Values given as the number of neurpspheres formed per 1000 cells.

### Serial MRI of cerebral ischaemia following FePro-NPC injection

**Figure 4.**
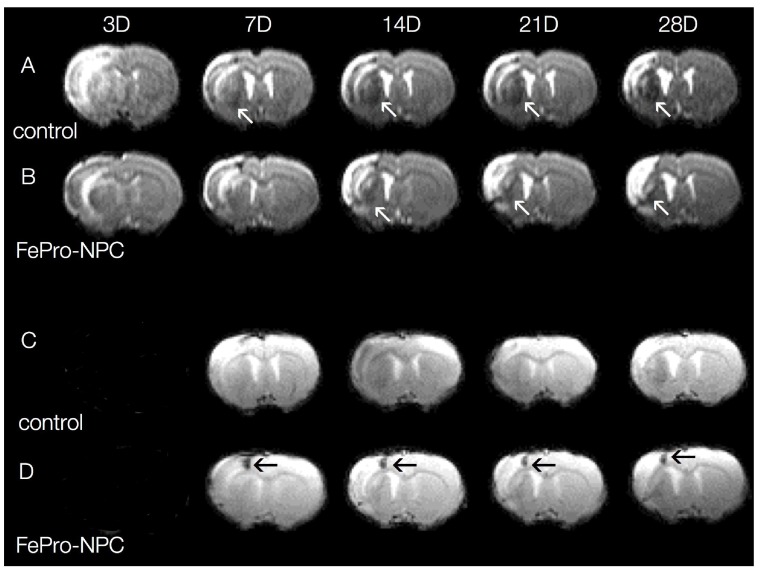
*In vivo* MRI. **A**, **B**, Serial T _2_-weighted imaging up to 28 days post-ischaemia in FePro-NPC and control animals. Regions of T _2_ hypointensity develop in the lesion at later timepoints (white arrows). **C**, **D**, Serial T _2_*-weighted imaging up to 28 days post-ischaemia in FePro-NPC and control animals. The injection site of FePro-labelled cells can be identified in the FePro-NPC group at all timepoints (black arrows).

*In vivo* MRI of injected FePro-labelled NPC was carried out 3, 7, 14, 21 and 28 days after cerebral ischaemia, to determine whether cells could be identified and monitored over time, relative to a control MCAO group without NPCs ( Figure 4). The T _2_-weighted images revealed extensive heterogeneous ischaemic lesions in both groups, with hypointense regions developing in the striatum and middle cortical layers, from day 7 and persisting until day 28 ( Figure 4A,B). This lesion heterogeneity was present in all control animals as well as in the FePro-NPC group. The FePro-labelled cells at the injection site were clearly detected in T _2_*-weighted images ( Figure 4C,D).

**Figure 5.**
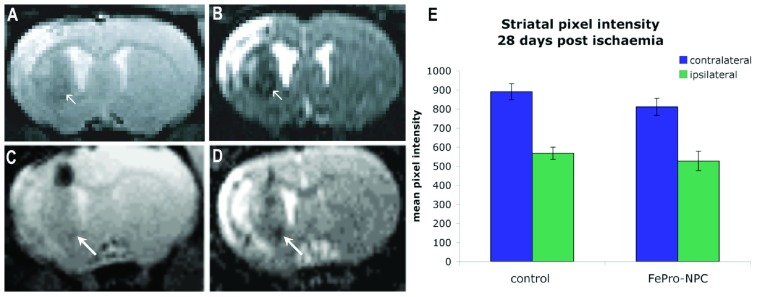
Hypointensity at 28 days post-ischaemia. T _2_- ( **B**, **D**) and T _2_*-weighted ( **A**, **C**) images are shown. **A**, **B**, MCA region in one control. **C,D**. MCA region in one FePro-NPC animal at 28 days post-ischaemia. Hypointense regions within the ischaemic lesion (arrows) were observed in both T _2_- and T _2_*-weighted images. **E**, Signal intensity in regions of T _2_-weighted hypointense ipsilateral striatum at 28 days post-ischaemia, and in corresponding contralateral regions in control (green) and FePro-NPC (blue) groups. Signal intensity in the ischaemic striatum was lower than in the contralateral striatum. Signal intensity was significantly different between contralateral and ipsilateral hemispheres (p<0.001).

We compared T _2_- and T _2_*-weighted images at 28 days in the FePro-NPC and control group. Figure 5A–D shows the profile of a FePro-NPC-treated animal and control animal at the 28 day time period. Lesion heterogeneity was observed in all animals in both T _2_ and T _2_*-weighted images. Signal intensity in hypointense ischaemic striatum regions was significantly different from the intensity in corresponding contralateral regions ( Figure 5E; F=3.154, p=0.05).

**Figure 6.**
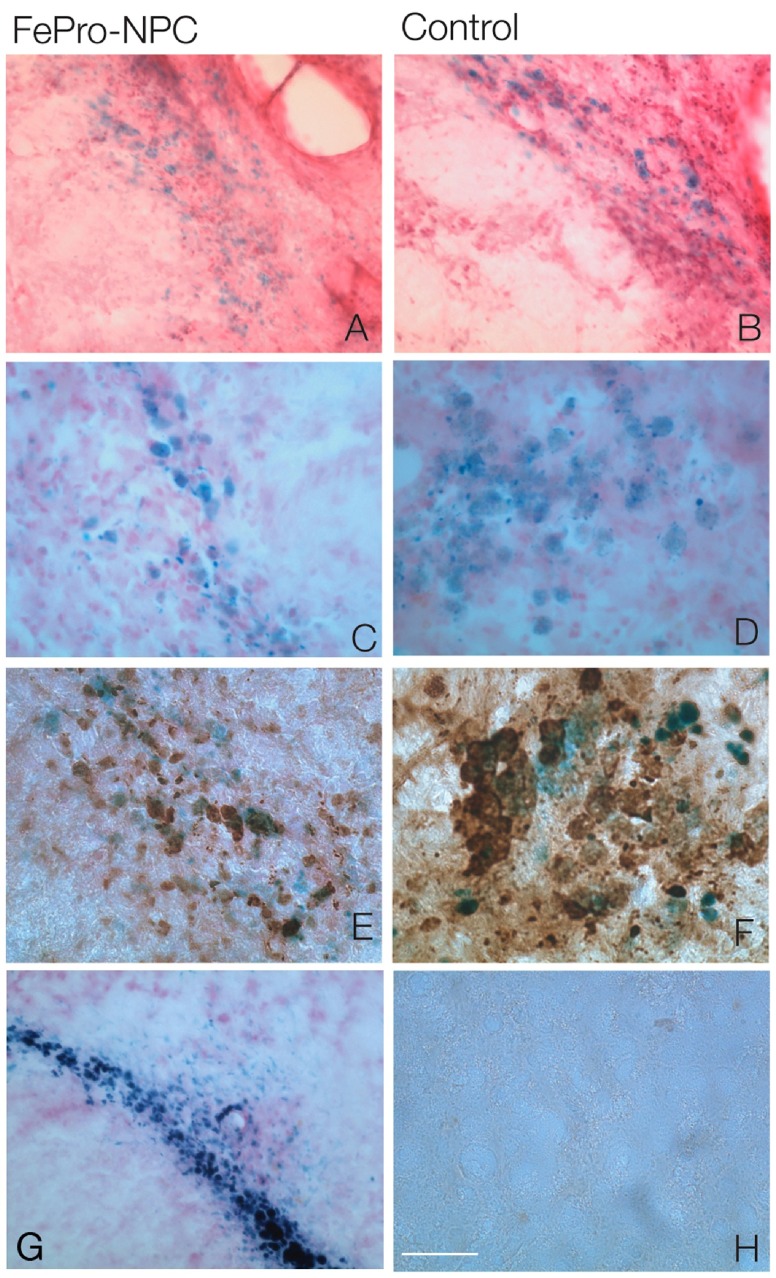
Histological analysis at 28 days at the ipsilateral striatum. **A**, **B**, Prussian blue stain for iron and hematoxylin-eosin staining at the ipsilateral striatum. Iron detected in the ischaemic lesion. **C**, **D**, Prussian blue stain with nuclear fast red counterstain in the ipsilateral striatum. **E**, **F**, OX-42 immunohistochemistry stain for macrophage/microglia, and Prussian blue stain for iron. Prussian blue-positive and – negative macrophage/microglia, detected by OX-42, are present at the ischaemic lesion. **G**, Prussian blue stain at the injection site in FePro-NPC ipsilateral hemisphere. **H**, OX-42 immunohistochemistry for macrophage/microglia and Prussian blue stain in the contralateral hemisphere. No macrophage/microglia or iron were observed in the striatum or cortex of the contralateral hemisphere. Scale bars, 30 µm.

We investigated the origin of the hypointense signal on T _2_-weighted MRI by examining histological sections. Iron can be a source of T _2_-weighted hypointensity, and we assessed its distribution in both groups. In both groups, iron was detected in the ipsilateral (ischaemic) striatum at the ischaemic border, and in the ischaemic cortex in some animals (2 of 5 control, and 3 of 6 FePro-NPC animals)( Figure 6C–F). Iron accumulation was detected in the mid-striatum at the lesion border, which approximates the area of hypointensity on MRI. Additionally in the FePro-NSC group, iron was detected at the injection site ( Figure 6G). No iron was detected in the contralateral cortex or striatum in the FePro-NSC group ( Figure 6H).

We investigated whether the MRI signal hypointensity was associated with the distribution of macrophage/microglia in the brain. We observed accumulated macrophage/microglia in the infarcted cortex and striatum in both FePro-NSC and control groups, and some of them co-labelled with iron ( Figure 6E,F). Iron-positive cells were a mixture of macrophage and non-macrophage cell types. No macrophage/microglia were observed in the contralateral hemispheres ( Figure 6G,H).

**Figure 7.**
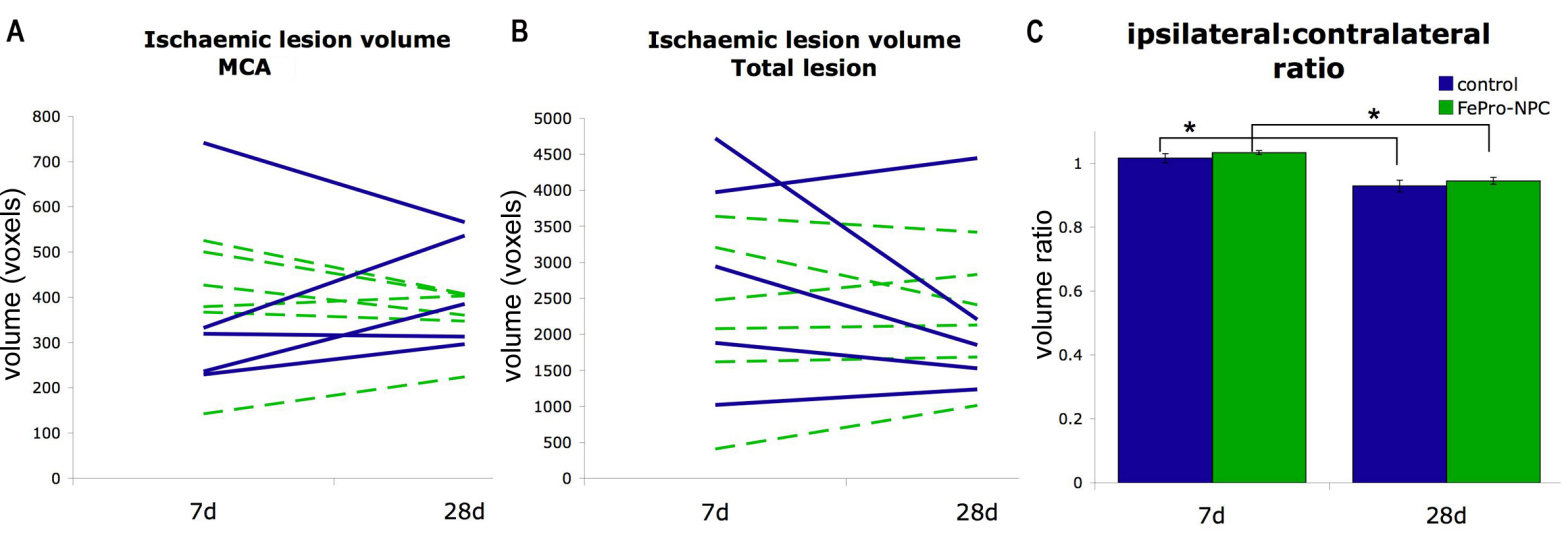
Analysis of lesion volume change 7 days and 28 days after MCAO in FePro-NPC grafted and controls. **A**, Lesion volume (cm ^3^/mL) of the central MCA region slice. There is no significant difference between controls (solid line, blue) and FePro-NPC (dashed line, green) lesion volumes at the MCA region (p=0.577). **B**, Total lesion volume. There is no significant difference between control and FePro-NPC lesion volumes (p=0.921). **C**, Hemisphere volume change. Change in ipsilateral:contralateral hemisphere volume ratio between 7 and 28 days (*:ANOVA, p<0.0001), but there is no significant difference between control and FePro-NPC in hemisphere volume.

Finally to investigate the effect of cell injections on the outcome of cerebral ischaemia, MRI analysis was carried out. Changes in volume of the lesion at the level of the central MCA region and the volume of the whole lesion over time were compared between control ischaemic and FePro-NPC groups. No statistically significant difference in lesion volume over time was observed either for the whole lesion or at the central MCA territory ( Figure 7A,B; p=0.275 and p=0.244, respectively). The change in hemisphere volume at the MCA territory was also assessed. We observed that the ipsilateral hemisphere volume was decreased by 28 days post-ischaemia relative to the contralateral hemisphere in both groups (F=17.18; p<0.005; Figure 7C), but we did not observe a difference between FePro-NPC and control groups. This reduction in ipsilateral hemisphere volume over time may represent secondary, delayed neuronal death in the ischaemic hemisphere, lesion compaction, brain reorganisation or a combination of these factors.

## Discussion

The main findings of this study were that i) FePro displays a high labelling efficiency and does not affect NPC behaviour *in vitro*; ii) following brain ischemia, the evolution of endogenous MRI T _2_-weighted contrast and heterogeneity can interfere with NPC detection and iii) soluble cues from injured tissue promote NPC migration *in vitro*, but no discernible NPC migration is observed *in vivo* following an extensive stroke.

### FePro displays high labelling efficiency and does not affect NPC behaviour

We observed that NPC can be stably labelled with FePro with no significant effect on cell viability, metabolic activity or proliferation relative to control, unlabelled cells, and that labelled NPC can differentiate into neurons, oligodendrocytes and astrocytes. The FePro label is retained throughout multiple cell divisions during the formation of new neurospheres and their differentiation. This is consistent with our previous *in vivo* labelling and tracking of endogenous stem cells that showed the presence of doublecortin-FePro-labelled cells 28 days after FePro injection into the lateral ventricle ^24^. In addition, data from the ST14A cell line ( Supplementary Figure 1) also confirm that FePro does not affect cell viability, and has greater labelling efficiency than Endorem. Imaging NPC *in vitro* demonstrated that FePro uptake under the described labelling conditions was sufficient to generate MRI contrast. SQUID measurement of iron oxide uptake also confirmed and quantified cellular uptake of FePro and suggest that FePro may be a superior MRI contrast agent for NPC labelling compared with Endorem ^24^. Our results indicate that there are no cytotoxic effects associated with FePro labelling, which is consistent with other studies that have similarly shown limited cytotoxicity of FePro in other cell types, and that FePro uptake is sufficient to generate contrast on T _2_*-weighted images ^20, 21, 33, 34^. Taken together with our previous work demonstrating that endogenous neural stem cells could be labelled with FePro and migrate from the subventricular zone (SVZ) to the olfactory bulb ^22^, NPCs are ideally suited for labelling with FePro for *in vivo* detection of cell migration.

### Evolution of MRI T _2_ contrast and heterogeneity interferes with NPC detection in ischaemic brain

This study demonstrated that cellular imaging was in part complicated by the evolution of T _2_ and T _2_* heterogeneity in the ischaemic lesion in all animals, obscuring cell detection on MRI at later timepoints. The evolution of T _2_-weighted hypointensity could be related to the accumulation of macrophages and granulocytes to the lesion over time, leading to localized hypercellularity. Accumulation of MHC Class II-expressing cells (such as macrophages, dendritic and B cells) was shown to be spatially coincident with regions of T _2_-weighted hypointensity and reduced ADC (apparent diffusion coefficient) in a model of multiple sclerosis ^35^. Hypercellularity was shown to increase from one week, peak at 3 weeks and persist in later timepoints, and regions of T _2_-weighted hypointensity develop by 3 weeks, which is consistent with our results ^35^. In another study, T _2_ hypointensity was observed from 4 days post-ischaemia at the lesion border, which interfered with detection of SPIO-labelled injected cells, and accumulation of ferric iron was detected histologically from 6 days post-ischaemia around the lesion ^36^. Although we did not observe haemorrhage in any of the animals, the T _2_ heterogeneity may also arise as a consequence of the accumulation of iron in the lesion, through the development of microhaemorrhages and deposition of haemosiderin ^37^, the degradation of blood products, or neuronal degeneration.

In the FePro-NSC group, an additional factor is clearly the presence of iron oxide particles in the FePro contrast agent. Palweczyk *et al.* have shown that iron exchange can occur between labelled cells and macrophages *in vitro*, although the transfer of iron to macrophages is low ^38^. Weber *et al.* observed the evolution of hypointensity in ischaemic striatum on T _2_*-weighted MRI at 2 and 10 weeks post-ischaemia in a MCA occlusion model, and found that T _2_* hypointense regions colocalised with iron-containing macrophages ^39^. The authors attributed the iron in macrophages to the phagocytosis of red blood cells from damaged blood vessels. Danielisova *et al.* have observed iron deposition in the striatum and pyramidal cells of cortical layers III and V following 20 minute MCA occlusion ^40^. Justicia *et al.* have reported iron accumulation, T _2_-weighted hypointensity and delayed neuronal death in the thalamus between 3 and 7 weeks following cerebral ischaemia ^41^ and suggested that iron accumulation was mediated by heme oxygenase-1-positive (HO-1) microglial activity. These studies suggest that iron accumulation at lesion sites may be linked to microglial activity and neurodegeneration.

Whereas our previous study has shown in normal brains a good correlation between MRI and FePro-labelled endogenous neural progenitor cells ^24^, tracking FePro-labelled cells in the ischaemic brain is clearly more challenging. There are a few studies that have transplanted iron-oxide or gadolinium labeled ES or NSC cells into the contralateral, non-ischaemic hemisphere (rather than in the ipsilateral stroked hemisphere), and observed migration of cells towards the ischaemic lesion ^19, 42– 44^. In these studies, transplanted cells were capable of migration via the corpus callosum into the ishaemic hemisphere, and could be visualised using MRI. However, it should be noted that T2 and T2* heterogeneity in the ischaemic lesion evolves over time, and it clearly becomes an issue particularly at later time points. This study highlights the need to characterise the long-term profile of cerebral ischaemia, and points to confounding image contrast at later timepoints that must be overcome in order for iron oxide-based cellular imaging to be a viable method of cell tracking.

### Soluble cues from injured tissue promote NPC migration *in vitro*

The NPC demonstrated a migratory response to cerebral ischaemia cues *in vitro*. These results indicate that fetal NPC are responsive to cerebral ischaemia factors, and the migratory cue is likely to be a soluble extracellular factor(s). To investigate the factors present in ischaemic tissue was beyond the scope of this study. However we tested factors that had been previously reported to be up-regulated following cerebral ischaemia, and to be associated with enhanced neurogenesis and neuroblast migration in our *in vitro* migration assays. We found that neurospheres were capable of migration in response to FGF-2 and EGF but did not migrate extensively in the presence of VEGF (data not shown). In contrast to the NPC migratory response observed *in vitro*, we did not observe any major cell migration from the injection site to the ischaemic striatum or cortex. It is possible that the soluble cues promoted cell migration *in vitro* because of the absence of inhibitory or toxic molecules present in the *in vivo* environment. It is also conceivable that the FePro-labelled NPCs were phagocytosed by macrophages *in vivo* ^45^. The apparent lack of accumulation of NPCs at the infarcted site correlates with the lack of a significant reduction in infarct size. These findings are consistent with a recent report showing that reduction in infarct size is “dose-dependent” and that neural stem cell injection is effective in reducing neural damage only following a moderate infarct ^46^. On the other hand, behavioural improvement following injection of NPC has been reported in the absence of a significant reduction in infarct size, and might be attributed to increased plasticity in the grafted brains, possibly via paracrine mechanisms as reported for both neural and non-neural stem cells ^16, 47– 49^.

In our study, NPCs were engrafted at 48 hours post-ischaemia. Data from the *in vitro* NPC migration assay demonstrated that NPCs responded to migratory cues from this time point, except at the highest concentration. In a recent study, Darsalia *et al.* demonstrated that this time point was optimal for cell survival and migration in a similar 30 minute occlusion model and intrastriatal engraftment of human fetal NPCs ^3^. However, engraftment at 48 hours post-ischaemia may not be the optimal time point to expect to have an effect on infarct volume. Therefore, functional assays would be important in future studies to establish a therapeutic effect of cell engraftment and cell labelling with MRI contrast agents. One study reported that engraftment of cells labelled with a gadolinium compound reduced functional recovery compared to engraftment of cells labelled with a fluorescent dye only ^50^. Cell migration has been observed in the corpus callosum in other studies engrafting murine embryonic stem cells or human NPC at later timepoints in models of cerebral ischaemia. Injection at the corpus callosum allows for cell injection at a distance from the lesion, reducing exposure to potentially toxic inflammatory environment, while providing a potential pathway for migration towards the lesion site and subsequent migration into the lesion. Clearly, cell type and origin, engraftment timepoint, engraftment site and infarct size are important factors for survival and migration of engrafted cells in ischaemic environments, as well as the parameters used to assess recovery.

## Conclusion

We have shown that the MRI cell tracking agent FePro does not affect NPC viability, proliferation or differentiation capacity *in vitro*. However, in the *in vivo* studies we have identified possible sources of T _2_-weighted image contrast in the ischaemic lesion that develops over time, creating a challenge for longitudinal cellular imaging studies. In conclusion, this study raises important issues surrounding the use of MRI and MRI contrast agents for longitudinal cell tracking studies in models of injury, where the evolution of endogenous contrast over time within lesioned tissue can be a source of uncertainty in image interpretation.

## List of abbreviations

ES cell – embryonic stem cell

GFAP – glial fibrillary acidic protein

IgG – immunoglobulin G

IgM – immunoglobulin M

MCA – middle cerebral artery

SQUID – superconducting quantum interference device

MTT – 3-[4,5-dimethylthiazol-2-yl]-2,5-diphenyl tetrazolium bromide

PFA – paraformaldehyde

SPIO – superparamagnetic iron oxide

## References

[1] Garcia JH Ye ZR: Epidemiology and pathology of occlusive cerebrovascular disease. In: Crockard A, Hayward R,Hoff JT (eds). *Neurosurgery. The scientific basis of clinical practice*, vol. 2. Blackwell Scientific: Oxford,2000;pp 704–726

[2] Arvidsson A Collin T Kirik D: Neuronal replacement from endogenous precursors in the adult brain after stroke. *Nat Med.* 2002;8(9):963–70 10.1038/nm747 12161747

[3] Darsalia V Allison SJ Cusulin C: Cell number and timing of transplantation determine survival of human neural stem cell grafts in stroke-damaged rat brain. *J Cereb Blood Flow Metab.* 2011;31(1):235–42 10.1038/jcbfm.2010.81 20531461 PMC3049487

[4] Kokaia Z Lindvall O: Neurogenesis after ischaemic brain insults. *Curr Opin Neurobiol.* 2003;13(1):127–32 10.1016/S0959-4388(03)00017-5 12593991

[5] Lindvall O Kokaia Z: Stem cells for the treatment of neurological disorders. *Nature.* 2006;441(7097):1094–6 10.1038/nature04960 16810245

[6] Aarum J Sandberg K Haeberlein SL: Migration and differentiation of neural precursor cells can be directed by microglia. *Proc Natl Acad Sci U S A.* 2003;100(26):15983–8 10.1073/pnas.2237050100 14668448 PMC307679

[7] Bang OY Lee JS Lee PH: Autologous mesenchymal stem cell transplantation in stroke patients. *Ann Neurol.* 2005;57(6):874–82 10.1002/ana.20501 15929052

[8] Kondziolka D Wechsler L: Stroke repair with cell transplantation: neuronal cells, neuroprogenitor cells, and stem cells. *Neurosurg Focus.* 2008;24(3–4):E13 10.3171/FOC/2008/24/3-4/E12 18341389

[9] Savitz SI Dinsmore J Wu J: Neurotransplantation of fetal porcine cells in patients with basal ganglia infarcts: a preliminary safety and feasibility study. *Cerebrovasc Dis.* 2005;20(2):101–7 10.1159/000086518 15976503

[10] Darsalia V Allison SJ Cusulin C: Cell number and timing of transplantation determine survival of human neural stem cell grafts in stroke-damaged rat brain. *J Cereb Blood Flow Metab.* 2011;31(1):235–42 10.1038/jcbfm.2010.81 20531461 PMC3049487

[11] Abrous DN Koehl M Le Moal M: Adult neurogenesis: from precursors to network and physiology. *Physiol Rev.* 2005;85(2):523–69 10.1152/physrev.00055.2003 15788705

[12] Darsalia V Kallur T Kokaia Z: Survival, migration and neuronal differentiation of human fetal striatal and cortical neural stem cells grafted in stroke-damaged rat striatum. *Eur J Neurosci.* 2007;26(3):605–14 10.1111/j.1460-9568.2007.05702.x 17686040

[13] Ishibashi S Sakaguchi M Kuroiwa T: Human neural stem/progenitor cells, expanded in long-term neurosphere culture, promote functional recovery after focal ischemia in Mongolian gerbils. *J Neurosci Res.* 2004;78(2):215–23 10.1002/jnr.20246 15378509

[14] Kelly S Bliss TM Shah AK: Transplanted human fetal neural stem cells survive, migrate, and differentiate in ischemic rat cerebral cortex. *Proc Natl Acad Sci U S A.* 2004;101(32):11839–44 10.1073/pnas.0404474101 15280535 PMC511061

[15] Lee HJ Kim KS Park IH: Human neural stem cells over-expressing VEGF provide neuroprotection, angiogenesis and functional recovery in mouse stroke model. *PLoS One.* 2007;2(1):e156 10.1371/journal.pone.0000156 17225860 PMC1764718

[16] Smith EJ Stroemer RP Gorenkova N: Implantation site and lesion topology determine efficacy of a human neural stem cell line in a rat model of chronic stroke. *Stem Cells.* 2012;30(4):785–96 10.1002/stem.1024 22213183

[17] Takahashi K Yasuhara T Shingo T: Embryonic neural stem cells transplanted in middle cerebral artery occlusion model of rats demonstrated potent therapeutic effects, compared to adult neural stem cells. *Brain Res.* 2008;1234:172–82 10.1016/j.brainres.2008.07.086 18703033

[18] Zhu W Mao Y Zhou LF: Reduction of neural and vascular damage by transplantation of VEGF-secreting neural stem cells after cerebral ischemia. *Acta Neurochir Suppl.* 2005;95:393–7 10.1007/3-211-32318-X_80 16463888

[19] Hoehn M Kustermann E Blunk J: Monitoring of implanted stem cell migration *in vivo*: a highly resolved *in vivo* magnetic resonance imaging investigation of experimental stroke in rat. *Proc Natl Acad Sci U S A.* 2002;99(25):16267–72 10.1073/pnas.242435499 12444255 PMC138600

[20] Zhang ZG Jiang Q Zhang R: Magnetic resonance imaging and neurosphere therapy of stroke in rat. *Ann Neurol.* 2003;53(2):259–63 10.1002/ana.10467 12557295

[21] Daadi MM Li Z Arac A: Molecular and magnetic resonance imaging of human embryonic stem cell-derived neural stem cell grafts in ischemic rat brain. *Mol Ther.* 2009;17(7):1282–91 10.1038/mt.2009.104 19436269 PMC2835224

[22] Arbab AS Yocum GT Kalish H: Efficient magnetic cell labeling with protamine sulfate complexed to ferumoxides for cellular MRI. *Blood.* 2004;104(4):1217–23 10.1182/blood-2004-02-0655 15100158

[23] Arbab AS Yocum GT Rad AM: Labeling of cells with ferumoxides-protamine sulfate complexes does not inhibit function or differentiation capacity of hematopoietic or mesenchymal stem cells. *NMR Biomed.* 2005;18(8):553–9 10.1002/nbm.991 16229060

[24] Panizzo RA Kyrtatos PG Price AN: *In vivo* magnetic resonance imaging of endogenous neuroblasts labelled with a ferumoxide-polycation complex. *Neuroimage.* 2009;44(4):1239–46 10.1016/j.neuroimage.2008.10.062 19059485

[25] Guzman R Uchida N Bliss TM: Long-term monitoring of transplanted human neural stem cells in developmental and pathological contexts with MRI. *Proc Natl Acad Sci U S A.* 2007;104(24):10211–6 10.1073/pnas.0608519104 17553967 PMC1891235

[26] Cattaneo E Conti L: Generation and characterization of embryonic striatal conditionally immortalized ST14A cells. *J Neurosci Res.* 1998;53(2):223–34 10.1002/(SICI)1097-4547(19980715)53:2<223::AID-JNR11>3.0.CO;2-7 9671979

[27] Kyrtatos PG Lehtolainen P Junemann-Ramirez M: Magnetic tagging increases delivery of circulating progenitors in vascular injury. *JACC Cardiovasc Interv.* 2009;2(8):794–802 10.1016/j.jcin.2009.05.014 19695550

[28] Lee KM Santos-Ruiz L Ferretti P: A single-point mutation in FGFR2 affects cell cycle and Tgfbeta signalling in osteoblasts. *Biochim Biophys Acta.* 2010;1802(3):347–55 10.1016/j.bbadis.2009.11.006 20004243

[29] Young KM Fogarty M Kessaris N: Subventricular zone stem cells are heterogeneous with respect to their embryonic origins and neurogenic fates in the adult olfactory bulb. *J Neurosci.* 2007;27(31):8286–96 10.1523/JNEUROSCI.0476-07.2007 17670975 PMC6331046

[30] Sommer I Schachner M: Monoclonal antibodies (O1 to O4) to oligodendrocyte cell surfaces: an immunocytological study in the central nervous system. *Dev Biol.* 1981;83(2):311–27 10.1016/0012-1606(81)90477-2 6786942

[31] Bansal R Warrington AE Gard AL: Multiple and novel specificities of monoclonal antibodies O1, O4, and R-mAb used in the analysis of oligodendrocyte development. *J Neurosci Res.* 1989;24(4):548–57 10.1002/jnr.490240413 2600978

[32] Badin RA Modo M Cheetham M: Protective effect of post-ischaemic viral delivery of heat shock proteins *in vivo*. *J Cereb Blood Flow Metab.* 2009;29(2):254–63 10.1038/jcbfm.2008.106 18781161 PMC2702130

[33] Arbab AS Bashaw LA Miller BR: Intracytoplasmic tagging of cells with ferumoxides and transfection agent for cellular magnetic resonance imaging after cell transplantation: methods and techniques. *Transplantation.* 2003;76(7):1123–30 10.1097/01.TP.0000089237.39220.83 14557764

[34] Arbab AS Liu W Frank JA: Cellular magnetic resonance imaging: current status and future prospects. *Expert Rev Med Devices.* 2006;3(4):427–39 10.1586/17434440.3.4.427 16866640

[35] Broom KA Anthony DC Blamire AM: MRI reveals that early changes in cerebral blood volume precede blood-brain barrier breakdown and overt pathology in MS-like lesions in rat brain. *J Cereb Blood Flow Metab.* 2005;25(2):204–16 10.1038/sj.jcbfm.9600020 15678123

[36] Vandeputte C Thomas D Dresselaers T: Characterization of the inflammatory response in a photothrombotic stroke model by MR: implications for stem cell transplantation. *Mol Imaging Biol.* 2011;13(4):663–71 10.1007/s11307-010-0395-9 20700767

[37] Kwa VI Franke CL Verbeeten B Jr: Silent intracerebral microhemorrhages in patients with ischemic stroke. Amsterdam Vascular Medicine Group. *Ann Neurol.* 1998;44(3):372–7 10.1002/ana.410440313 9749604

[38] Pawelczyk E Arbab AS Chaudhry A: *In vitro* model of bromodeoxyuridine or iron oxide nanoparticle uptake by activated macrophages from labeled stem cells: implications for cellular therapy. *Stem Cells.* 2008;26(5):1366–75 10.1634/stemcells.2007-0707 18276802

[39] Weber R Wegener S Ramos-Cabrer P: MRI detection of macrophage activity after experimental stroke in rats: new indicators for late appearance of vascular degradation?. *Magn Reson Med.* 2005;54(1):59–66 10.1002/mrm.20532 15968679

[40] Danielisova V Gottlieb M Nemethova M: The effect of preconditioning on the iron deposition after transient forebrain ischemia in rat brain. *Arch Ital Biol.* 2004;142(2):87–94 15248564

[41] Justicia C Ramos-Cabrer P Hoehn M: MRI detection of secondary damage after stroke: chronic iron accumulation in the thalamus of the rat brain. *Stroke.* 2008;39(5):1541–7 10.1161/STROKEAHA.107.503565 18323485

[42] Modo M Cash D Mellodew K: Tracking transplanted stem cell migration using bifunctional, contrast agent-enhanced, magnetic resonance imaging. *Neuroimage.* 2002;17(2):803–11 10.1006/nimg.2002.1194 12377155

[43] Modo M Mellodew K Cash D: Mapping transplanted stem cell migration after a stroke: a serial, *in vivo* magnetic resonance imaging study. *Neuroimage.* 2004;21(1):311–7 10.1016/j.neuroimage.2003.08.030 14741669

[44] Obenaus A Dilmac N Tone B: Long-term magnetic resonance imaging of stem cells in neonatal ischemic injury. *Ann Neurol.* 2011;69(2):282–91 10.1002/ana.22168 21387373 PMC3069664

[45] Detante O Valable S de Fraipont F: Magnetic resonance imaging and fluorescence labeling of clinical-grade mesenchymal stem cells without impacting their phenotype: study in a rat model of stroke. *Stem Cells Transl Med.* 2012;1(4):333–41 10.5966/sctm.2011-0043 23197812 PMC3659696

[46] Daadi MM Hu S Klausner J: Imaging Neural Stem Cell Graft-Induced Structural Repair in Stroke. *Cell Transplant.* 2013;22(5):881–92 10.3727/096368912X656144 23044338 PMC5823270

[47] Andres RH Horie N Slikker W: Human neural stem cells enhance structural plasticity and axonal transport in the ischaemic brain. *Brain.* 2011;134(Pt 6):1777–89 10.1093/brain/awr094 21616972 PMC3102243

[48] Patkar S Tate R Modo M: Conditionally immortalised neural stem cells promote functional recovery and brain plasticity after transient focal cerebral ischaemia in mice. *Stem Cell Res.* 2012;8(1):14–25 10.1016/j.scr.2011.07.001 22099017

[49] Prasongchean W Bagni M Calzarossa C: Amniotic fluid stem cells increase embryo survival following injury. *Stem Cells Dev.* 2012;21(5):675–88 10.1089/scd.2011.0281 21905920

[50] Modo M Beech JS Meade TJ: A chronic 1 year assessment of MRI contrast agent-labelled neural stem cell transplants in stroke. *Neuroimage.* 2009;47(Suppl 2):T133–42 10.1016/j.neuroimage.2008.06.017 18634886 PMC4145694

