# Supplementary material for: Monitoring ferumoxide-labelled neural progenitor cells and lesion evolution by magnetic resonance imaging in a model of cell transplantation in cerebral ischaemia
Source: F1000Res. 2014 Mar 4;2:252. Originally published 2013 Nov 21. [Version 2] doi: 10.12688/f1000research.2-252.v2 (PMC3962009; doi:10.12688/f1000research.2-252.v2)
Supplement: Peer review history

# Peer review: Referee response for version 1

**Published:** 2014-01-21

**Authors:** Keith Muir

**Affiliations:** 1 Institute of Neuroscience and Psychology, University of Glasgow, Glasgow, UK

This study highlights difficulties with the use of MRI contrast agents for the imaging of cells in vivo in stroke models.

The evolution of ischaemic damage after stroke typically involves a degree of petechial haemorrhage with deposition of haemosiderin commonly evident histologically or on sensitive MRI sequences after an iscaemic event. Confounding of the signal from iron-labelled cell therapies is therefore likely, as described here.

In vitro functional assays offer some evidence that the MRI contrast agent may not affect important properties of the NPC cell line, but the in vivo assessment is incomplete and deserves comment. Effects on infarct volume are highly unlikely with therapies delivered 48h after ischaemia, so this is unsurprising and neither supports nor argues against a therapeutic effect. Functional assays are more difficult and time consuming, but a rodent focal ischaemia model with delayed cell delivery would offer the only means of establishing whether any therapeutic effect is compromised by the cell label. This would be important for further study since it has previously been reported that intracellular MRI labels (a gadolinium conjugate) negated functional benefits from a neural stem cell line (Modo *et al.* Neuroimage. 2009;47 Suppl 2:T133-42).

I have read this submission. I believe that I have an appropriate level of expertise to confirm that it is of an acceptable scientific standard.

# Peer review: Referee response for version 1

**Published:** 2013-11-21

**Authors:** Roger Barker

**Affiliations:** 1 Department of Clinical Neurosciences, University of Cambridge, Cambridge, UK

This project is investigating the migration and effects of rat neural precursor cells on ischaemic brain lesions using an improved method for labelling these cells in vivo using an iron based marker. The authors show that the label does not interfere with the normal behaviour of the cells, and that it allows for cells to be seen in the grafted brain. However the study also highlights that the cells have no benefit on the lesion (when grafted 48 hours after the lesion) and in addition as the vascular lesion re-organises, the iron signal can be hard to see over endogenous iron deposition.

This is a straightforward study with clear data, my only comments are:

1. What is the efficiency of uptake on the iron label into cells, in terms of how many of the cells take up the label?
2. How stable is the iron label within the cells in vitro? Is it lost over time independent of any cell death- namely are the number of cells with a label the same over time in the stem cell cultures?
3. What factors present in the ischemic tissue facilitate NPC migration?
4. It would be of interest to know if transplanting the cells into the non stroked hemisphere would have allowed for better visualisation of cell migration to the lesion.

I have read this submission. I believe that I have an appropriate level of expertise to confirm that it is of an acceptable scientific standard.
